# The Interplay of HIV and Autophagy in Early Infection

**DOI:** 10.3389/fmicb.2021.661446

**Published:** 2021-04-28

**Authors:** Romina Cabrera-Rodríguez, Silvia Pérez-Yanes, Judith Estévez-Herrera, Daniel Márquez-Arce, Cecilia Cabrera, Lucile Espert, Julià Blanco, Agustín Valenzuela-Fernández

**Affiliations:** ^1^Laboratorio de Inmunología Celular y Viral, Unidad de Farmacología, Sección de Medicina, Facultad de Ciencias de la Salud, e IUETSPC de la Universidad de La Laguna, Campus de Ofra s/n, Tenerife, Spain; ^2^AIDS Research Institute IrsiCaixa, Institut de Recerca en Ciències de la Salut Germans Trias i Pujol (IGTP), Barcelona, Spain; ^3^Institut de Recherche en Infectiologie de Montpellier, Université de Montpellier, CNRS, Montpellier, France; ^4^Universitat de Vic-Central de Catalunya (UVIC-UCC), Catalonia, Spain

**Keywords:** HIV-1, autophagy, Env signaling, early infection, cell-death

## Abstract

HIV/AIDS is still a global threat despite the notable efforts made by the scientific and health communities to understand viral infection, to design new drugs or to improve existing ones, as well as to develop advanced therapies and vaccine designs for functional cure and viral eradication. The identification and analysis of HIV-1 positive individuals that naturally control viral replication in the absence of antiretroviral treatment has provided clues about cellular processes that could interact with viral proteins and RNA and define subsequent viral replication and clinical progression. This is the case of autophagy, a degradative process that not only maintains cell homeostasis by recycling misfolded/old cellular elements to obtain nutrients, but is also relevant in the innate and adaptive immunity against viruses, such as HIV-1. Several studies suggest that early steps of HIV-1 infection, such as virus binding to CD4 or membrane fusion, allow the virus to modulate autophagy pathways preparing cells to be permissive for viral infection. Confirming this interplay, strategies based on autophagy modulation are able to inhibit early steps of HIV-1 infection. Moreover, autophagy dysregulation in late steps of the HIV-1 replication cycle may promote autophagic cell-death of CD4^+^ T cells or control of HIV-1 latency, likely contributing to disease progression and HIV persistence in infected individuals. In this scenario, understanding the molecular mechanisms underlying HIV/autophagy interplay may contribute to the development of new strategies to control HIV-1 replication. Therefore, the aim of this review is to summarize the knowledge of the interplay between autophagy and the early events of HIV-1 infection, and how autophagy modulation could impair or benefit HIV-1 infection and persistence, impacting viral pathogenesis, immune control of viral replication, and clinical progression of HIV-1 infected patients.

## Introduction

### A General Overview of HIV: Factors Related to the Control of the Infection and Pathogenesis

According to the Global Human Immunodeficiency Virus (HIV) and Acquired Immune Deficiency Syndrome (AIDS) statistics report published in 2020 by UNAIDS (2020), there were around 38 million people living with HIV and 1.7 million new cases in 2019, and about 26 million people had access to the antiretroviral therapy (ART) at the end of June 2020. Most new infections and deaths are caused by HIV-1 subtype. Advances in science, accumulated experience in the field, along with active community and political commitment, mean that the public health threat of the HIV pandemic could be over by 2030, but there are still many social, economic and healthcare obstacles and inequalities to overcome (Colvin, 2011; UNAIDS, 2015; Poku, 2016). Due to active ART and associated medical achievements, the scientific community considers HIV/AIDS a chronic disease, mainly when access to effective care is assured (Forsyth and Valdiserri, 2012; McGrath et al., 2014).

Regardless of the above-mentioned achievements, ART does not eliminate the virus from latent reservoirs (Chun et al., 1997, 1998; Finzi et al., 1997; Perelson et al., 1997; Wong et al., 1997; Chun and Fauci, 1999; Davey et al., 1999; Hermankova et al., 2003; Siliciano and Greene, 2011; Rosenbloom et al., 2017), such as lymph nodes, gut associated lymphoid tissue (GALT), the central nervous system (CNS) or the most recent discovery of persistence in adipose tissue (Couturier and Lewis, 2018; van Marle et al., 2018). Latent reservoirs favor the emergence of new drug resistances which affect treatment adherence. Moreover, as HIV patients need long-term medication, metabolic concerns, tissue damage and associated aging emerge as a challenge for new drugs and ART regimes (Chawla et al., 2018; Gu et al., 2020). Thus, promising results have been obtained with pre-exposure prophylaxis (PrEP) strategies, which aim to prevent both HIV-1 infection and transmission and are highly dependent on patient adherence (Straubinger et al., 2019), or by using broadly neutralizing antibodies (bnAbs) that could achieve prolonged viral suppression by directly targeting different epitopes from key viral proteins and activating the immune response in order to remove Ab-targeted infected cells (Carrillo et al., 2018; Kumar et al., 2018; Grobben et al., 2019).

HIV-1 entry and early infection is mediated when the virus interacts with a main receptor in target cells, CD4, and a co-receptor, mainly CCR5 or CXCR4 which are involved in HIV-1 pathogenesis (Dalgleish et al., 1984; McDougal et al., 1985, 1986; Cocchi et al., 1995, 1996; Alkhatib et al., 1996; Bleul et al., 1996; Choe et al., 1996; Deng et al., 1996; Doranz et al., 1996, 1999; Dragic et al., 1996; Liu et al., 1996; Samson et al., 1996; Aramori et al., 1997; Davis et al., 1997; Farzan et al., 1997; Weissman et al., 1997; Blanco et al., 2000; Brelot et al., 2000; Clapham and McKnight, 2001; Chanel et al., 2002; Moore et al., 2004; Tuttle et al., 2004; Cavarelli et al., 2008; Cavarelli and Scarlatti, 2011; Gorry and Ancuta, 2011; Wilen et al., 2012; Melikyan, 2014). It should be mentioned that an effective cure for HIV-1 infection has been reported in two patients by *CCR5*Δ*32/*Δ*32*-hematopoietic stem cells (HSCs) transplantation, observing that latently infected cells were eliminated and replaced with HIV-1 resistant donor CCR5^–/–^ cells (Hütter et al., 2009; Gupta et al., 2019). Although it is not possible to universally apply this method (Kordelas et al., 2014), it shows that HIV-1 can be eradicated from the organism (Hütter et al., 2009; Allers et al., 2011; Allers and Schneider, 2015; Haworth et al., 2017; Ambinder et al., 2020). In addition, a plethora of different strategies to keep the latent reservoir under control have been developed, from gene therapy to therapeutic vaccines (Rerks-Ngarm et al., 2009; Elliott et al., 2014; Henrich et al., 2014, 2017; Rasmussen et al., 2014; Luzuriaga et al., 2015; Søgaard et al., 2015; Zhu et al., 2015; Bar et al., 2016; Kaminski et al., 2016a, b; Kuritzkes, 2016; Scheid et al., 2016; Wang et al., 2016; Colby et al., 2018; Davenport et al., 2019).

The relationship between CCR5 or more precisely the lack of CCR5 expression and autophagy in the context of HIV-1 infection is not clear enough. It has been reported that HIV-1 envelope (Env) complex of proteins that targets cells through the cell-surface CCR5 receptor (i.e., R5-tropic Envs) triggers autophagy and cell death in bystander uninfected CD4^+^ T cells, after long-term virus-cell contacts (Blanco et al., 2004; Espert et al., 2009). However, this autophagy cell death process is triggered by gp41-induced membrane fusion, and is independent of the co-receptor usage (Espert et al., 2009). Additionally, the role of CCR5 seems to differ in CD4^+^ T cells and monocyte-differentiated macrophages (MDMs). CD4^+^ T cells are susceptible to cell death induced by CXCR4 and CCR5 using viral strains and MDMs infected by CCR5-using viruses show weak autophagy activation and increased viral replication compared to MDMs infected with viral strains that use CXCR4 (Espert et al., 2009). This is consistent with the fact that CCR5-tropic viral strains are more infectious than CXCX4-tropic viral strains in MDMs (Schweighardt et al., 2004; Willey et al., 2005; Petrov et al., 2013; Wang et al., 2015; Bolduc et al., 2017; Quitadamo et al., 2018; Borrajo et al., 2019), and with the *in vivo* prevalence of viruses that utilize CCR5 in the majority of infected individuals (De Jong et al., 1992; Roos et al., 1992; Schuitemaker et al., 1992; Connor et al., 1993; Zhu et al., 1993; van’t Wout et al., 1994; Cornelissen et al., 1995; Spijkerman et al., 1995; Huang et al., 1996; Reece et al., 1998; Keele and Derdeyn, 2009; Salazar-Gonzalez et al., 2009). Likewise, functional CCR5 reduced the expression of autophagy genes, such as *BECN1*, *ATG5* and *microtubule associated protein 1 light chain 3 (LC3) alpha* (*MAP1LC3A*), and promoted inflammation in an experimental asthmatic mouse model (Liu et al., 2021). Hence, an antagonist peptide of CCR5 enhances the mRNA levels of *BECN1*, *ATG5*, and *MAP1LC3A* and their proteins level, as well as double-membrane autophagic vesicles or vacuoles (AVs), thereby decreasing inflammation (Liu et al., 2021). These AVs are considered hallmarks of autophagy (Arstila and Trump, 1968; Punnonen et al., 1989; Dunn, 1990a, b; Liou et al., 1997; Mizushima et al., 2002; Zhou et al., 2016), the autophagosomes. Therefore, it is plausible that the lack of CCR5 expression could be associated with a fully active autophagy process presenting a protective phenotype against X4-tropic viral strains. Thus, functional CCR5 appears to be involved in autophagy inhibition during HIV infection with R5-tropic strains. However, HIV-1 mediated cell-death autophagy by long-term non-infectious HIV-1 Env/cell contacts is independent of CCR5 (Espert et al., 2009). Therefore, the CCR5/HIV-1/autophagy interplay and its role in viral life cycle is a complex and active subject of research (reviewed in Espert et al., 2008).

One noteworthy discovery in the HIV-1 pandemic history is the existence of infected individuals that naturally control the viral replication for longer than 10 years in the absence of ART, known as long-term non-progressors (LTNP; Lambotte et al., 2005). Briefly, depending on the clinical progression of the disease, there are different groups of LTNP HIV^+^ individuals: the elite controllers (LTNP-EC), with HIV-1 RNA plasma levels below the level of detection (less than 50 copies of HIV-1 RNA/mL), viremic controllers (LTNP-VC) with HIV-1 RNA levels equal to or below 2,000 copies/mL, non-controllers (LTNP-NC) with levels above 2,000 copies/mL and viremic non-progressors (VNP) with more than 10,000 copies/mL and normal levels of CD4^+^ T lymphocytes (Lambotte et al., 2005; Diop et al., 2006; Deeks and Walker, 2007; Canducci et al., 2009; Grabar et al., 2009; Lajoie et al., 2009; Limou et al., 2009; Okulicz et al., 2009; Casado et al., 2010, 2018; Rotger et al., 2010; Zhang et al., 2010; Gurdasani et al., 2014; Cabrera-Rodriguez et al., 2019). Nevertheless, despite being controllers, there is evidence of ongoing viral replication in LTNP-EC (Blankson et al., 2007; Lamine et al., 2007; Dinoso et al., 2008; Migueles et al., 2008; Pereyra et al., 2008, 2009; Hatano et al., 2009; Julg et al., 2010; Mens et al., 2010; O’Connell et al., 2010; Salgado et al., 2010; Zaunders et al., 2011; Noel et al., 2016; Ali et al., 2018; Casado et al., 2018; Woldemeskel et al., 2020), and this endorses the importance of periodically monitoring these patients. The causes of this viral replication control are diverse and may be due to both host and viral factors, thereby making it difficult to establish a precise cause for the natural control of HIV. The viral factors include replicative capacity, infectivity, tropism, virulence, mutation rate, viral load or genetic variants in different genes. This is the case of the Australian cohort characterized by invalidating alterations in the *nef* gene or the Spanish LTNP-C cluster with a heritable conserved non-functional *env* gene, which is responsible for the low binding of the viral Env complex to CD4, inefficient cell-signaling and subsequent impaired fusion, infection and viral transmission capacities (Zaunders et al., 2011; Casado et al., 2018; Lopez-Galindez et al., 2019; López-Galíndez, 2019). Likewise, LTNP phenotypes are usually associated with genetic factors in the host related to immune control in viral replication and reservoir generation (Pereyra et al., 2010; Poropatich and Sullivan, 2011; Whittall et al., 2011; Buckheit et al., 2012; Bullen et al., 2014; Colomer-Lluch et al., 2018; Goulder and Deeks, 2018; Lopez-Galindez et al., 2019). HLA alleles are well known genetic markers associated with protection or progressive disease, but *CCR5*-Δ32 allelic deletions or forkhead box O3a (FOXO3) mutations are also linked with the survival of memory CD4^+^ T cells (de Roda Husman et al., 1997; Eugen-Olsen et al., 1997; Pasi et al., 2000; Dabrowska et al., 2008; van Grevenynghe et al., 2008; Casado et al., 2010; López-Galíndez, 2019). Cell antiviral restriction factors are a first line of defense against viral infection, replication and spreading, such as the tripartite motif-containing protein 5 alpha isoform (TRIM5α), SAM and HD domain containing deoxynucleoside triphosphate triphosphohydrolase 1 (SAMHD1), tetherin or BST2 (bone marrow stromal cell antigen 2), apolipoprotein B mRNA editing enzyme, catalytic polypeptide-like (APOBEC) proteins or histone deacetylase 6 (HDAC6; Valenzuela-Fernandez et al., 2005; Valera et al., 2015; Soliman et al., 2017; Marrero-Hernández et al., 2019). In this regard, proautophagic HDAC6 has recently been reported to be closely linked to HIV-1 control (Valenzuela-Fernandez et al., 2005, 2008; Valera et al., 2015; Casado et al., 2018; Marrero-Hernández et al., 2019), similar to other proteins implicated in the autophagy process (Rotger et al., 2011; Nardacci et al., 2014, 2017), opening up a new pathway to control HIV-1 infection. Thus, recent data are addressed below that indicate that non-functional *env*-gene of viruses from LTNP-EC individuals is responsible for a defect in Env-mediated productive signaling to overcome the barrier of HDAC6, an autophagy-related factor which blocks HIV-1 fusion, transmission and early infection (Casado et al., 2018). Therefore, the capacity of primary viral Env to signal and overcome this proautophagic restriction factor, at the early stage of the viral life cycle, is related to the progressor phenotype of HIV^+^ patients (Casado et al., 2018; Cabrera-Rodriguez et al., 2019), whereas non-functional viral Envs are associated with viruses of HIV^+^ individuals presenting the LTNP-EC phenotype (Casado et al., 2018).

### Autophagy and HIV

Autophagy is a complex and orchestrated degradative process that maintains cell homeostasis, eliminating misfolded/old elements and recycling them to obtain nutrients, and is also relevant in the innate and adaptive immunity against pathogen infection, including viruses (Liang et al., 1998, 2014; Meléndez et al., 2003; Pinan-Lucarré et al., 2003; Gutierrez et al., 2004; Nakagawa et al., 2004; Dengjel et al., 2005; Ogawa et al., 2005; Paludan et al., 2005; Birmingham et al., 2006; Hara et al., 2006; Komatsu et al., 2006, 2007; Jounai et al., 2007; Kohda et al., 2007; Lee et al., 2007; Nakai et al., 2007; Nishiyama et al., 2007; Py et al., 2007; Nedjic et al., 2008; Dupont et al., 2009; English et al., 2009; Fujita et al., 2009; Masiero et al., 2009; Yamaguchi et al., 2009; Zhang et al., 2009b; Lee H.K. et al., 2010; Liu et al., 2010; Orvedahl et al., 2010; Ponpuak et al., 2010; Travassos et al., 2010; Levine et al., 2011; Benjamin et al., 2013; Randow and Youle, 2014; Sagnier et al., 2015; Valera et al., 2015; Dasari et al., 2016; Hu et al., 2016; Paul and Münz, 2016; Staring et al., 2017; Viret et al., 2018; Oh and Lee, 2019; Dong et al., 2021).

In mammalian cells autophagosome formation is initiated by signals that activate the mammalian autophagy-related 1 (ATG1) homologs, unc-51 like autophagy activating kinase 1 (ULK1) and ULK2 (Kuroyanagi et al., 1998; Yan et al., 1998, 1999; Tomoda et al., 1999; Young et al., 2006; Chan et al., 2007; Kundu et al., 2008; Egan et al., 2011; Chan, 2012). The mammalian ULK complex is formed by the serin/threonin protein kinase ULK1 and ULK2, ATG13, ATG101, and RB1CC1 (RB1-inducible coiled-coil protein 1) (named FIP200, focal adhesion kinase family interacting protein of 200 kDa) (Hosokawa et al., 2009; Jung et al., 2009; Mercer et al., 2009; Noda and Fujioka, 2015; Qi et al., 2015). This complex initiates the formation of the phagophore, a shaped double membrane structure, which elongates to engulf cytoplasmic components, and finally closes to form an autophagosome (Hara et al., 2008; Chan, 2009; Nakatogawa et al., 2009; Yang and Klionsky, 2010; Weidberg et al., 2011b). The ULK complex is then located in phagophores (Hara et al., 2008) where it regulates the initial events of autophagosome formation together with the class III phosphatidylinositol 3-kinase catalytic subunit type 3 (PIK3C3) complex, consisting of BECN1 (named beclin 1 or ATG6), ATG14 (also named ATG14L), PIK3C3 (named VPS34), and PIK3R4 (phosphoinositide-3-kinase regulatory subunit 4) (named VPS15) (Hara et al., 2008; Itakura et al., 2008; Liang et al., 2008; Ganley et al., 2009; Hosokawa et al., 2009; Jung et al., 2009; Matsunaga et al., 2009; Mercer et al., 2009; Zhong et al., 2009; Yang and Klionsky, 2010). Of note, the ULK complex is negatively regulated by the mechanistic target of rapamycin complex 1 or mammalian target of rapamycin complex 1 (MTORC1, named mTORC1)-mediated phosphorylation of ULK1, ULK2, and ATG13 (Ganley et al., 2009; Hosokawa et al., 2009; Jung et al., 2009), whereas the activity of the BECN1-PIK3C3 complex could be regulated by its interaction with several cofactors, such as UVRAG (UV radiation resistance associated), ATG14 and AMBRA1 (autophagy and beclin 1 regulator 1) (Matsunaga et al., 2009; Zhong et al., 2009; Di Bartolomeo et al., 2010; Mizushima et al., 2011). The elongation of the phagophore needs the recruitment of different factors, such as the WD-repeat protein interacting with phosphoinositides (WIPI) family proteins (yeast ATG18 homologs), ATG2 and the transmembrane protein ATG9 (Lang et al., 2000; Jeffries et al., 2004; Proikas-Cezanne et al., 2004; Young et al., 2006; Obara et al., 2008; Longatti and Tooze, 2009; Polson et al., 2010; Mauthe et al., 2011; Dooley et al., 2014; Grimmel et al., 2015; Gómez-Sánchez et al., 2018; Osawa et al., 2019; Tang et al., 2019; Bozic et al., 2020; Matoba et al., 2020; Sawa-Makarska et al., 2020). The ubiquitin-like (Ubl) conjugation reaction is also required for elongation of the phagophore and involves the conjugation of ATG12 to ATG5 which recruits autophagy related 16 like 1 (ATG16L1) (previous name ATG16L) (Mizushima et al., 1999, 2003; Kuma et al., 2002; Hanada et al., 2007; Walczak and Martens, 2013; Fahmy and Labonté, 2017; Harada et al., 2019; Karow et al., 2020). Furthermore, this complex is able to associate with ATG8 family proteins, thereby allowing ATG8 proteins to conjugate to phosphatidylethanolamine on the phagophore membrane (Nakatogawa et al., 2007). In mammals, there are at least seven ATG8 family members, such as MAP1LC3A and MAP1LC3B, with two isoforms of MAP1LC3B: gamma-aminobutyric acid receptor-associated protein (GABARAP) and GABARAP like 1 and 2 (GABARAPL1 and GABARAPL2) (Xin et al., 2001; He et al., 2003; Weidberg et al., 2010, 2011a; Shpilka et al., 2011; Shvets et al., 2011). Both MAP1LC3A/B and GABARAP subfamily members are conjugated to the phagophore (Kabeya et al., 2000, 2004) and required for the formation of the autophagosome (Weidberg et al., 2010). Additionally, ULK1, ATG13, and RB1CC1 (members of the UKL1 complex) have been reported to interact with members of the MAP1LC3A/B family of proteins (Alemu et al., 2012; Suzuki et al., 2014). Then, phosphorylation of several of these factors by ULK1 initiates the nucleation process and autophagosome biogenesis (Ganley et al., 2009; Hosokawa et al., 2009; Jung et al., 2009). The outer membrane of autophagosomes then fuses with a lysosome allowing lysosomal hydrolytic enzymes to degrade the intra-autophagosomal components together with the inner membrane. Finally, selective autophagy clearance of different cargos is driven by their association with several factors that are recruited to the inner surface of the phagophore via their LC3-interacting region (LIR) motifs, such as sequestosome 1 (SQSTM1)/p62, NBR1 [neighbor of breast cancer 1 (NBR1) autophagy cargo receptor; also named neighbor of BRCA1 gene 1] and OPTN (optineurin) (Pankiv et al., 2007; Ichimura et al., 2008; Kirkin et al., 2009; Wild et al., 2011).

Furthermore, as introduced above, autophagy could act as a first innate immune barrier against invading viruses, clearing them, and also being part of the immune response by means of antigen presentation by major histocompatibility complex (MHC) or human leukocyte antigen (HLA) class I and class II molecules (Brazil et al., 1997; Nimmerjahn et al., 2003; Dengjel et al., 2005; Dörfel et al., 2005; Paludan et al., 2005; Zhou et al., 2005; Zwart et al., 2005; Schmid et al., 2007; Li et al., 2008; Gannage and Münz, 2010; Tey and Khanna, 2012; Bell et al., 2013; Valečka et al., 2018). In this respect, different research studies have tried to develop vaccines in order to promote immune presentation of viral proteins through the autophagy degradation pathway (Andersen et al., 2016). However, autophagy/virus interplay is complex as pathogens have evolved strategies to inhibit or use autophagy to their own benefit. Thus, this process can be either favoring or blocking viral replication (Lee et al., 2007; Denizot et al., 2008; Zhou and Spector, 2008; Espert et al., 2009; Joubert et al., 2009; Kyei et al., 2009; Blanchet et al., 2010; Grégoire et al., 2011, 2012; Levine et al., 2011; Bhatt and Damania, 2012; Chaumorcel et al., 2012; Campbell and Spector, 2013; Liang et al., 2013; Richetta et al., 2013; Jackson, 2015; Valera et al., 2015; Mailler et al., 2019; Mao et al., 2019; Marrero-Hernández et al., 2019; Chen et al., 2020).

Autophagy has become relevant in HIV-1 infection, since it is clearly involved in the late stages of the viral life cycle (i.e., replication and viral egress), preventingviral infection in CD4^+^ T cells and favoring viral replication in macrophages (Zhou and Spector, 2008; Espert et al., 2009; Kyei et al., 2009; Valera et al., 2015; Mailler et al., 2019; Marrero-Hernández et al., 2019; Chen et al., 2020).

The sections below describe scientific data concerning the role of autophagy in the first steps of HIV-1 infection, presented from two points of view: (i) the ability of the virus to modulate autophagy during viral entry and infection and (ii) the consequences of autophagy modulation, in target cells, for early HIV-1 infection.

## The Role of Autophagy in the Early Steps of HIV-1 Infection

The HIV-1 life cycle could be divided into early and late stages, defined by the integration of viral genome into host cells. The early (pre-integration) steps of the infection begin with the attachment of the virion at the cell-surface and end with the integration of the proviral DNA into the host genome (Arhel, 2010; Campbell and Hope, 2015; Deeks et al., 2015; Goodsell, 2015). The late stages (post-integration) initiate with proviral transcription, assembly of viral components at cell-surface, viral egress, and maturation (Stevenson, 2003; Sutton et al., 2013; Deeks et al., 2015; Freed, 2015; Goodsell, 2015; Mailler et al., 2016). The HIV life cycle could end with cell death of infected cells or by HIV-1 Env-mediated cell death of uninfected bystander cells (Mohammadi et al., 2013; Cummins and Badley, 2014; Doitsh and Greene, 2016; Li et al., 2020) which occurs during early infection (Espert et al., 2008, 2009). Hence, one of the most relevant HIV-1 modulators of cell death is the Env glycoprotein (Krüger et al., 1996; Blanco et al., 2001; Vlahakis et al., 2001; Garg and Blumenthal, 2006, 2008; Varbanov et al., 2006; Garg et al., 2009; Sivaraman et al., 2009; Gorry and Ancuta, 2011; Cicala et al., 2016; Joshi et al., 2016; Tsao et al., 2016; Evering, 2018; Daussy et al., 2020).

The HIV-1 *env*-gene encodes the Env glycoprotein precursor (gp160), which, upon cleavage by the furin cellular protease, results in the generation of the gp120 surface and the gp41 transmembrane glycoproteins that assemble in trimer to form the active Env complex (Decroly et al., 1994, 1997; Moulard et al., 1999; Staropoli et al., 2000; Checkley et al., 2011; Wilen et al., 2012; Chung et al., 2014; Melikyan, 2014; Ward and Wilson, 2017; Schneck et al., 2020). During HIV-1 entry, the viral surface gp120 subunit of the Env binds to CD4 and CCR5 or CXCR4 key co-receptor (Dalgleish et al., 1984; McDougal et al., 1985, 1986; Cocchi et al., 1995, 1996; Alkhatib et al., 1996; Bleul et al., 1996; Choe et al., 1996; Deng et al., 1996; Doranz et al., 1996, 1999; Dragic et al., 1996; Liu et al., 1996; Samson et al., 1996; Aramori et al., 1997; Davis et al., 1997; Farzan et al., 1997; Weissman et al., 1997; Blanco et al., 2000; Brelot et al., 2000; Clapham and McKnight, 2001; Chanel et al., 2002; Moore et al., 2004; Tuttle et al., 2004; Cavarelli et al., 2008; Cavarelli and Scarlatti, 2011; Gorry and Ancuta, 2011; Wilen et al., 2012; Colin et al., 2013, 2018; Melikyan, 2014; Shaik et al., 2019). Hence, HIV-1 Env is responsible for virus attachment to target cells, membrane fusion and subsequent genome-bearing capsid entry into the cytoplasm, thereby initiating the productive infection process (Markosyan et al., 2003; Chang et al., 2005; Dobrowsky et al., 2008; Wilen et al., 2012; Melikyan, 2014; Sood et al., 2017; Stultz et al., 2017; Iliopoulou et al., 2018; Chen, 2019). During these steps, HIV-1 Env-mediated signals prepare cells to be efficiently infected, where membrane dynamics and intracellular organelles and cytoskeleton reorganization could account for permissive control in early infection (Blanco et al., 2003; Jolly and Sattentau, 2004; Jolly et al., 2004; Valenzuela-Fernandez et al., 2005; Jimenez-Baranda et al., 2007; Barrero-Villar et al., 2008, 2009; Yoder et al., 2008; Liu et al., 2009; Puigdomenech et al., 2009; Garcia-Exposito et al., 2011, 2013; Stolp and Fackler, 2011; Vorster et al., 2011; de Armas-Rillo et al., 2016; Casado et al., 2018; Cabrera-Rodriguez et al., 2019). HIV-1 Env-induced autophagy would therefore be a good way to regulate early steps of the viral life cycle.

The interplay between early HIV-1 infection and autophagy is presented below by mainly focusing on the effects driven by the viral Env, and considering different studies performed in permissive cell types and/or tissues, such as T lymphocytes, peripheral blood mononuclear cells (PBMCs), macrophages and dendritic cells (DCs), and in the central nervous system (CNS).

### HIV-1 Env-Mediated Regulation of Autophagy and Related Factors During Early Infection in T Lymphocytes

Although HIV-1 induction of apoptosis in bystander CD4^+^ T cells was reported to be driven by Env glycoproteins, gp120 and gp41 (Blanco et al., 2003), Env-mediated autophagy during HIV-1 early infection was first described in 2006 (Espert et al., 2006). It was reported that Env-transfected or HIV-infected cells expressing the viral Env complex at the cell-surface induced autophagy and accumulation of BECN1 in uninfected CD4^+^ T lymphocytes via CXCR4 (Espert et al., 2006), where these autophagy events induce apoptosis in bystander CD4^+^ cells (Espert et al., 2006). The autophagic machinery is activated after long-lasting interaction of uninfected CD4^+^ T lymphocytes with Env expressed at the cell-surface of X4-tropic HIV-1 infected cells, thus accumulating the BECN1/ATG6 protein which regulates the mechanism interacting with PIK3 class III (Espert et al., 2006, 2007). These reports further highlighted that CD4 signaling and tyrosine-protein kinase Lck (LCK; named LCK proto-oncogene, Src family tyrosine kinase or p56^*lck*^) were not required and that autophagy was necessary for apoptosis to be induced. Moreover, CXCR4 or CCR5 signaling does not seem to be involved in HIV-1 Env-induced autophagy, which is dependent on the gp41 fusion capacity (Blanco et al., 2003; Denizot et al., 2008). These results suggest that HIV-mediated autophagic cell-death of bystander CD4^+^ T lymphocytes is a mechanism that could contribute to immunodeficiency.

It should also be mentioned that autophagy can be also induced by R5-tropic HIV-1 Env upon binding to bystander, uninfected CCR5^+^/CD4^+^ T cells, whereas it is inhibited in CD4^+^ T cells that are infected either by X4-tropic or R5-tropic viral strains (Molina et al., 2007).

Furthermore, after contact between CD4^+^ T lymphocytes and Env-expressing cells, reactive oxygen species (ROS) are accumulated and its production is known to be involved in autophagy (Molina et al., 2007; Borel et al., 2012). It has recently been reported that peroxisomes, organelles involved in the control of oxidative stress, are targeted by Env-mediated autophagy, inducing oxidative stress and this leads to uninfected CD4^+^ T cells death (Daussy et al., 2020).

Another aspect of HIV-1 infection and autophagy is that this retrovirus is also able to manipulate the process in order to survive. A new study in CD4^+^ T lymphocytes has shown that autophagy is rapidly controlled after HIV-1 entry by the viral protein R (Vpr) which reduces the amount of essential autophagy ATG proteins, such as MAP1LC3 and BECN1, and the autophagy-related protein BNIP3 (BCL2 interacting protein 3) and their mRNA, and is further implicated in the degradation of a transcription factor FOXO3 (Alfaisal et al., 2019).

### HIV-1 Env Modulates Autophagy and Related Factors Associated With Membrane and Cytoskeleton Dynamics, Key Events for Productive Early Infection

Autophagy is a paradigm of the cellular membrane dynamics driven by cytoskeleton components and other associated factors (Aplin et al., 1992; Fass et al., 2006; Axe et al., 2008; Nakatogawa et al., 2009; Hailey et al., 2010; Lee J.Y. et al., 2010; Mari et al., 2010; Ravikumar et al., 2010; Aguilera et al., 2012; Longatti and Tooze, 2012; Yamamoto et al., 2012; Ge et al., 2013; Knævelsrud et al., 2013; Puri et al., 2013; Zhuo et al., 2013; Coutts and La Thangue, 2015; Kast and Dominguez, 2015; Kast et al., 2015; Mi et al., 2015; de Armas-Rillo et al., 2016; Galluzzi et al., 2017a; Morel, 2020). Similarly, there are data that point to membrane dynamics being essential for successful HIV-1 entry and infection, but the mechanisms behind these events are complex and likely involve several factors. Thus, phosphatidylinositol (4,5)-bisphosphate (PIP_2_) is a second messenger that has been demonstrated to be important not only in the HIV-1 viral egress but also in the entry, synthetized by phosphatidylinositol-4-phosphate 5-kinase type Iα (PIP5K1A), after Env-gp120 interaction with CD4^+^ T lymphocyte (Barrero-Villar et al., 2008). Another cell factor that works in membrane dynamics is ADP ribosylation factor 6 (ARF6; Radhakrishna and Donaldson, 1997; Franco et al., 1999; Donaldson, 2003; Naslavsky et al., 2003; Aikawa and Martin, 2005; Donaldson and Honda, 2005), a GTPase which plays a role in the PIP_2_ generation (Honda et al., 1999; Toda et al., 1999; Brown et al., 2001; Santy and Casanova, 2001; Powner and Wakelam, 2002; Aikawa and Martin, 2005; Bach et al., 2010). ARF6 promotes autophagosome biogenesis, since it works favoring endocytic uptake of the plasma membrane into autophagosome precursors (Moreau et al., 2012), exerting a vital function for effective and productive viral entry (Garcia-Exposito et al., 2011).

In the context of cytoskeleton dynamics, actin adaptors and enzymes that reorganize, stabilize and sever cortical F-actin, such as MSN (moesin), FLNA (filamin A), and GSN (gelsolin) have been involved in early infection, and are responsible for pore fusion and viral entry (Jimenez-Baranda et al., 2007; Barrero-Villar et al., 2009; Garcia-Exposito et al., 2013). Tubulin cytoskeleton and associated factors also play a crucial role in driving membrane dynamics, as reported for cytoplasmic tubulin-deacetylase HDAC6 that controls pore fusion formation and viral entry (Valenzuela-Fernandez et al., 2005, 2008). Viral Env mediates acetylation of α-tubulin in order to allow a reorganization of the tubulin cytoskeleton and efficient pore fusion formation (Valenzuela-Fernandez et al., 2005; Casado et al., 2018). On the contrary, over-expression of HDAC6, which is located in the cytoplasm and regulates deacetylation of α-tubulin in stable microtubules (Hubbert et al., 2002; Valenzuela-Fernandez et al., 2008), inhibits HIV-1 Env-mediated tubulin acetylation, thereby preventing HIV-1 Env-dependent cell fusion and infection (Valenzuela-Fernandez et al., 2005).

Moreover, HDAC6 plays an important role in clearing accumulated protein aggregates, potentially toxic for cells by autophagy (Kawaguchi et al., 2003; Kopito, 2003). The C-terminal ubiquitin-binding ZnF-UBP zinc finger domain (ZnF-UBP or BUZ domain) of HDAC6 binds free ubiquitin as well as mono- and polyubiquitinated proteins with high affinity (Seigneurin-Berny et al., 2001; Hook et al., 2002; Kawaguchi et al., 2003; Boyault et al., 2006), facilitating the transport of ubiquitinated misfolded proteins to form an aggresome in a microtubule network dependent manner (Kawaguchi et al., 2003). In addition, HDAC6 is directly involved in autophagy by promoting the removal of aggresomes (Kawaguchi et al., 2003; Iwata et al., 2005; Pandey et al., 2007; Lee J.Y. et al., 2010). In fact, HDAC6 controls the fusion of autophagosomes and lysosomes by recruiting cortactin, and other key components for actin reorganization, thereby triggering clearance of autophagic substrates (Lee J.Y. et al., 2010). In an HIV-1 context, HDAC6 regulates infectivity of nascent HIV-1 virions by interacting with APOBEC subunit 3G (APOBEC3G; named A3G), stabilizing it and promoting the autophagic degradation of the HIV-1 infectivity factor Vif, thereby impairing the incorporation of Vif in nascent viral particles (Valera et al., 2015; Marrero-Hernández et al., 2019). Moreover, HDAC6 acts as a restriction factor, limiting viral production and infection by driving Pr55^*Gag*^ and Vif viral proteins to degradation through an aggresome/autophagy route (Marrero-Hernández et al., 2019). Thus, for HIV-1, targeting HDAC6 appears to be critical for assuring viral production and virus infectivity, and this could be a key proinfective function of Nef. Hence, HDAC6 is counteracted by functional Nef which drives its clearance by an acidic, endocytic/lysosomal pathway. In this regard, Nef assures viral production and infection by targeting HDAC6, stabilizing Pr55^*Gag*^ and Vif, thereby facilitating Pr55^*Gag*^ location and aggregation at the plasma membrane and subsequent virus production and infection capacity (Marrero-Hernández et al., 2019).

Therefore, the interplay between Nef and HDAC6 may be important to determine the course of HIV-1 infection and pathogenesis in infected individuals, and may contribute to the development of new strategies against HIV.

### The Ability of HIV-1 Env of Virus From Infected Individuals to Subvert or Not Autophagy-Associated Factors Is Related to the Patient Phenotype

It has recently been shown that inefficient HIV-1 Env functions and signaling may contribute to the low viral replication capacity and transmissibility of the viruses from natural controllers (LTNP-ECs) (Casado et al., 2013). Furthermore, the contribution of common viral features to the clinical LTNP-EC phenotype and a functional characterization of the initial events of the viral infection demonstrated that Envs from virus of LTNP-EC individuals were ineffective in the binding to CD4 and in the key triggering of actin/tubulin-cytoskeleton modifications, compared to Envs from viruses of HIV-1^+^ chronic patients (Casado et al., 2018). The viral properties of the cluster viruses result in defective viral fusion, entry, and infection, and these properties were inherited by every virus in the cluster (Casado et al., 2018). Along the same lines, another study that compared Env clones from virus of VNP patients with those from rapid progressors (RPs), revealed that both groups of primary viral Envs induced autophagy in bystander uninfected CD4^+^ T cells after 3 days of co-culture (Cabrera-Rodriguez et al., 2019). These results were demonstrated after long-lasting cell-to-cell experiments, and were entirely different to first HIV-1-cell contact and signaling to productive early infection (Casado et al., 2018), as this type of late transmission activates different mechanisms such as apoptosis and linked autophagy, leading to an increase in MAP1LC3 and cell-death (Espert et al., 2007; Cabrera-Rodriguez et al., 2019).

Related to the above deadly autophagy activation by HIV-1 Env, it has been reported that viral Env activates peroxisome degradation with a concomitant decrease in the expression of peroxisomal proteins [i.e., CAT (catalase) and PEX14 (peroxisomal biogenesis factor 14; also named peroxin 14)], which appears to be autophagy dependent, since down-regulation of BECN1 and SQSTM1/p62 restores their expression levels (Daussy et al., 2020). It is noteworthy that late autophagy occurrence is reported to be independent of the HIV-1 infected phenotype, VNP or RP, but it strongly correlated with the fusogenic capacity of viral Envs (Cabrera-Rodriguez et al., 2019). This means that functional viral Envs promote deadly late autophagy, under these experimental conditions, regardless of the HIV-1^+^ patients’ phenotype, and is directly related to the infectious capacity of the HIV-1 Envs. In this regard, inefficient primary HIV-1 Envs from LTNP-EC individuals are not able to signal and surpass the blockade exerted by the proautophagic, tubulin-deacetylase HDAC6 function. However, functional HIV-1 Envs from VNP or RP patients manage to signalize, overcoming the tubulin-deacetylase HDAC6 protective barrier and infect (Casado et al., 2018; Cabrera-Rodriguez et al., 2019). Therefore, this is the first evidence that a defect of the viral Env from HIV-1^+^ natural controllers to signal and neutralize the proautophagic factor HDAC6 is related to a defect in infection, virus variability and the LTNP-EC phenotype.

Regarding the above-mentioned ideas, the association between autophagy events, viremic control in HIV^+^ individuals and the progression of the disease suggests a new clinical perspective for HIV/AIDS. In this regard, it has been reported that LTNP and LTNP-EC phenotypes have PBMCs with a higher amount of AVs together with an increased expression of autophagic factors with respect to normal progressors (Nardacci et al., 2014). Additionally, this work shows that treatment of PBMC isolated from LTNP-EC individuals with rapamycin, an inhibitor of the MTOR, promotes autophagy, resulting in the impairment of viral production (Nardacci et al., 2014). Of note, in humanized mouse models, pan-inhibitors of the MTORC1 inhibit HIV-1 infection by interfering with virus entry, reducing CCR5 levels, and with transcription (Heredia et al., 2015). These data suggest that autophagy could contribute to the control of viral pathogenesis in HIV-1 viremic controllers (LTNP and LTNP-EC) and interfere with CCR5-mediated virus transmission, thereby constituting a potential target to drive the development of new therapies (Nardacci et al., 2014).

### Autophagy and Early HIV-1 Infection in Macrophages and Dendritic Cells

It is noteworthy that autophagy-associated cell-death is not similarly induced in uninfected macrophages, following exposure to viral particles (van Grol et al., 2010), despite being positive for the presence of autophagosomes (Espert et al., 2009), suggesting that Env-mediated autophagy is a cell-dependent process. However, it is important to bear in mind that during macrophage entry of the virus, other molecules are involved in the reorganization of membrane proteins necessary for HIV-1 entry, such as ceramides, integrins and heparan sulfate proteoglycan, with a demonstrated role of ceramides as inducers of autophagy that could eclipse the pro-autophagic functions of the HIV-1 Env/main receptor interplay (Finnegan et al., 2004; Borel et al., 2012). Furthermore, blockade of macrophage-autophagy appears to be a barrier for HIV-1, since viral replication is favored by inducing autophagy in infected macrophages (Kyei et al., 2009) and autophagy inhibition in infected macrophages abrogates viral replication (Espert et al., 2009; Kyei et al., 2009).

In DCs, HIV-1 induces a rapid shutdown of autophagy as HIV-1 Env activates the MTOR pathways, increasing cell-associated HIV-1 and transfer of the virus to CD4^+^ T cells and, thus, altering autophagy initiation leading to progressive autophagy exhaustion (Blanchet et al., 2010). In this regard, it has been demonstrated that fusion between endosomes and autophagosomes is inhibited during early HIV-1 infection by activation of MTOR, which is a negative regulator of autophagy (Altfeld et al., 2011). HIV-1 Env can activate the MTORC1 complex resulting in efficient viral replication and viral production. In fact, HIV-1 infection increases MTORC1 activity in both productively infected and bystander cells (Akbay et al., 2020).

On the contrary, it has been reported that autophagy is activated during HIV-1 infection in DCs. HIV-1 enters these cells by using C-type lectins as a receptor, and once inside the cell its RNA is recognized by toll-like receptors (TLRs), inducing the immune response and the beginning of the autophagy process. Although these events are under discussion, there is a clear-cut relationship between this viral recognition and the activation of the immune response together with the antiviral autophagy (Lee et al., 2007; Delgado et al., 2008; Shi and Kehrl, 2008; Blanchet et al., 2010; Altfeld et al., 2011; Nakamoto et al., 2012).

### Autophagy and HIV-1 Infection in the Central Nervous System

The CNS is also attacked by HIV-1 and, even under ART, at least half of HIV-1 positive individuals present associated neurocognitive disorders (HAND) with progressive neurotoxicity, neurodegeneration, and a chronic activation of the inflammatory response, but it is difficult to study and is a matter of controversy how HIV-1 manages to reach the CNS and how it infects different cell types (reviewed in Rojas-Celis et al., 2019). Autophagy is involved in both neuroprotection and neurodegeneration, but some research data support the hypothesis that the dysregulation of autophagy during HIV-1 infection is important in the pathogenesis of the neuropathology associated with AIDS.

At a more physiologically relevant level, it has been reported that the levels of several key autophagy factors, such as BECN1, ATG5, ATG7 and the membrane-associated MAP1LC3-phosphatidylethanolamine conjugate (named LC3-II) were higher in postmortem brains presenting HIV-1 encephalitis, compared with HIV-1 patients without encephalitis (Zhou et al., 2011). Additionally, these authors confirmed that overexpression of both X4- or R5-tropic gp120 increased the presence of these autophagy markers. The study further suggests that HIV-1 gp120 induces autophagy in neuron cells, and that the induction of autophagy might be related to the pathogenesis of neuroAIDS (Zhou et al., 2011).

Furthermore, a study carried out on primary human fetal astrocytes revealed that the HIV-1 Env subunit gp120 induced the expression of interleukin 6 (IL6) [named IFNB2 (interferon, beta 2)] through nuclear factor-kappa B (NFKB; named NF-κB) (Shah et al., 2011). This NFKB1-associated signal triggered the expression of some autophagy genes, such as *BECN1*, *ATG5*, and *MAP1LC3* whose proteins translocate from cytoplasm to the nucleus, and induce the phosphorylation of the inhibitory kappa B (IκB) factor (NFKB inhibitor), which promotes the autophagy degradative pathway (Copetti et al., 2009; Nivon et al., 2009, 2012; Zhang et al., 2009a; Romano et al., 2010; Jiang et al., 2011; Meng and Cai, 2011; Shah et al., 2011; Bhatnagar et al., 2012; Verzella et al., 2020). This event has also been reported in astrocytes, where HIV-1 gp120 increases CXCL8 (named IL8) production involving NFKB (Shah and Kumar, 2010). There have also been some works implicating HIV-1 gp120 in an increase of autophagy proteins, such as the MAP1LC3-phosphatidylethanolamine conjugate, ATG5, ATG7 and a downregulation of *MTOR*, whose phosphorylated form suppresses autophagy in the astrocyte cell line or in human fetal astrocytes (Cao et al., 2016). A recent study showed that the V3 loop region of HIV-1 gp120 is responsible for inducing autophagy in primary rat hippocampal neurons in a PRKA (protein kinase AMP-activated; named AMPK)/MTOR-dependent pathway (Liu et al., 2019). Moreover, if the autophagy induction is short term, it could inhibit apoptosis. On the contrary, if it lasts for a long time, it could play a detrimental role (Liu et al., 2019).

The relationship between HIV-1 Env and autophagy in the CNS seems to be highly important but little is known about the mechanisms behind the early phases of HIV-1 infection and more studies are needed to elucidate these questions (Rojas-Celis et al., 2019).

**FIGURE 1:**
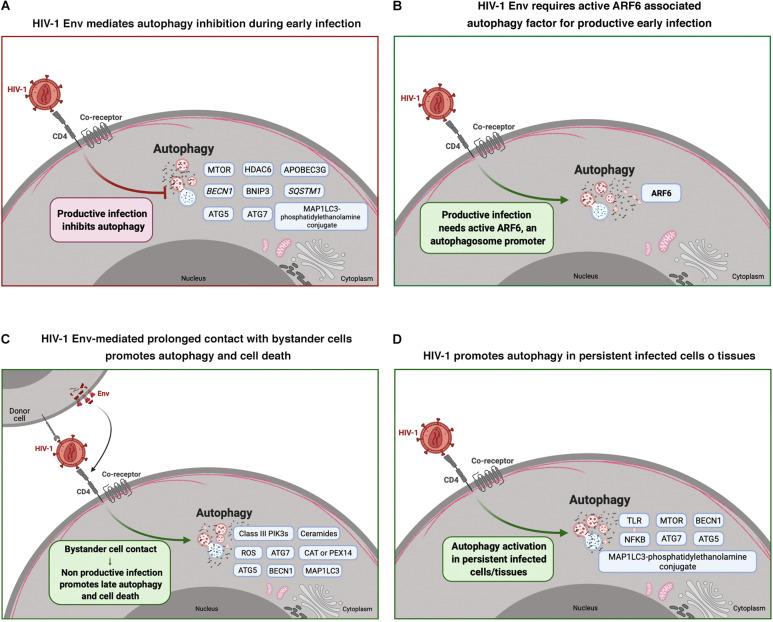
HIV-1 modulation of autophagy during early infection and viral non-productive bystander cell contact, and in persistent infected cells/tissues. Cell scheme summarizing the effect of HIV-1 on autophagy and associated factors in different HIV-1 scenarios. **(A)** HIV-1 Env mediates autophagy inhibition during early infection in CD4^+^ T cells. Autophagy associated factors affected by HIV-1 Env signaling are shown in blue boxes. **(B)** This scheme shows that HIV-1 Env requires active ARF6 associated autophagy factor for productive infection in CD4^+^ T cells. **(C)** This panel shows autophagy associated factors (blue boxes) that are activated during non-productive virus-bystander cell contacts. This HIV-1 Env-triggered late autophagy activation leads to CD4^+^ cell death. **(D)** This panel shows autophagy associated factors (in blue boxes) that are activated by HIV-1 infection in persistent infected cells and/or tissues. Arrow colors represent the reported positive (green) and negative (red) effects on the different steps of the viral life cycle, with dotted arrows signaling the HIV-1 activation pathway. This figure supports the information summarized in Table 1. Cell schemes were created with BioRender.com.

Therefore, autophagy is able to play a role as a modulator of HIV-1 infection at the early stages of its viral life cycle (summarized in Table 1 and Figure 1), but all these ideas and conclusions should be appropriately contextualized, since the use of a particular cell model or experimental procedure could condition the results obtained and conclusions drawn (reviewed in Killian, 2012; Leymarie et al., 2017). Taken together, HIV-1-mediated autophagy events could explain, at least in part, the pathophysiology of the viral disease.

**TABLE 1:** Summary of the HIV-1 effect on autophagy or associated factors.

	HIV-1 effect on autophagy or associated factors	
	Inhibition	Activation
**Productive early infection**	MTOR^2^, HDAC6, APOBEC3G, *SQSTM1/p62*, ATG5, ATG7, MAP1LC3-phosphatidylethanolamine conjugate, *BECN1*, BNIP3	ARF6
**Bystander cell death** (Late autophagy and cell death)		Class III PIK3s, ceramides^1^, ROS, peroxisomes proteins (CAT or PEX14), BECN1, ATG5, ATG7, MAP1LC3-phosphatidylethanolamine conjugate
**Infected cells/tissue**		TLR^2^, BECN1 ^3,A,B,C^ ATG5^3,A,B,C^, ATG7^3,A,B^, MAP1LC3-phosphatidylethanolamine conjugate^3,A,B,C^, MTOR^3,D^, NFKB^3,D^

*This table summarizes the effect of HIV-1 on autophagy and associated factors in different HIV-1 scenarios (indicated in left column): (i) during productive infection; (ii) by Env-mediated virus or infected cells contact with bystander target CD4^+^ cells; and (iii) in persistent infected cells and tissues. The modulatory effect of HIV-1 on autophagy or associated factors that are indicated refer to results obtained in CD4^+^ T lymphocytes, and when data have also been reported in other cell types and tissues, they are indicated as follows: ^1^Macrophages; ^2^DCs; ^3^studies in CNS. Regarding the latter, the data are indicated in terms of the studied cell/tissue as follows: ^A^Neuroblastoma cells, ^B^human brain tissue, ^C^human astrocytes and ^D^rat hippocampal neurons. This information is illustrated in Figure 1.*

## The Effect of Autophagy Modulation in the Early Steps of HIV-1 Infection

There are other strategies that have shed some light on the involvement of autophagy in HIV-1 early infection, such as the use of autophagy-chemical inhibitors, targeting the expression of autophagy-associated proteins by RNA interference (RNAi) or over-expressing its mutants, or by using the new clustered regularly interspaced short palindromic repeats (CRISPR)/Cas9 technology to abrogate their functions. The CRISPR/Cas system is the most recent genome-edition toolbox of much interest because of its ability to manipulate genomic sequences, enabling the creation of cell lines and animal models for the study of human diseases with promising clinical therapeutic applications, as well as in science and biotechnology (reviewed in Horvath and Barrangou, 2010; Marraffini and Sontheimer, 2010; Zhang et al., 2014; Hille et al., 2018; Leonova and Gainetdinov, 2020).

The inhibition of autophagy during active HIV-1 infection has been considered as a potential supplementary treatment to effective ART for controlling HIV-1 infection (Li et al., 2020).

There are diverse pharmacological ways to inhibit the autophagy process (Vakifahmetoglu-Norberg et al., 2015; Galluzzi et al., 2017b) and some of these have been assayed on HIV-1 infection. The chemical autophagy inhibitor 3-methyladenine (3-MA) that interferes with the PIK3 class III activity to impair autophagosome formation (Seglen and Gordon, 1982; Liang et al., 1999; Klionsky et al., 2008), prevents apoptosis in target CD4^+^ T cells exposed to Env-expressing effector cells, and monocyte-derived macrophage infection (Espert et al., 2009). Moreover, HDAC6-mediated pro-autophagic and anti-HIV-1 activity is abrogated by 3-MA, thereby stabilizing viral Pr55^*Gag*^ and Vif, assuring efficient viral production and infectiveness, respectively (Valera et al., 2015; Marrero-Hernández et al., 2019). This fact has been confirmed by using dead-mutants of HDAC6, neutralized in the deacetylase activity of the enzyme and deleted in the proautophagic BUZ C-terminal domain, which are both unable to promote autophagy degradation of these key viral proteins (Valenzuela-Fernandez et al., 2005; Valera et al., 2015; Marrero-Hernández et al., 2019).

The use of constructs that directly encode mutants of proteins, as previously seen with MAP1LC3 (Borel et al., 2015), or indirectly implicated in autophagy membrane dynamics provides some indicators for developing possible strategies against HIV-1 infection. For example, and taking together some of the ideas described above, the expression of kinase-dead mutant D227A (D/A)-PIP5K1A, reduces PIP_2_ production during first virus-cell contacts impairing HIV-1 infection (Barrero-Villar et al., 2008), and perturbing ARF6/PIP_2_-plasma membrane bio-regeneration, by using non-functional ARF6 mutants, abrogates HIV-1 Env/CD4-driven viral entry and infection (Garcia-Exposito et al., 2011). An interesting strategy to control autophagy and affect HIV-1 infection has been developed by using Tat-Beclin 1 construct, which is able to interact with the auxiliary viral factor Nef, that inhibits HIV-1 replication in primary human macrophages (Shoji-Kawata et al., 2013).

Bafilomycin A1 (Baf A1) is another candidate to analyze the effect of autophagy inhibition on HIV-1 infection. Baf A1 is an inhibitor of vacuolar H^+^ ATPases [V-type ATPases (ATP6V)] that blocks acidification of the lysosome and its fusion with autophagosomes, thus inhibiting autophagic degradation (Yamamoto et al., 1998; Klionsky et al., 2012). It has been reported that Baf A1 prevents HDAC6-mediated autophagy degradation of viral Pr55^*Gag*^ and Vif proteins and stabilizes SQSTM1/p62 protein (Marrero-Hernández et al., 2019), which is a key autophagic factor that fades during active autophagy (Ichimura et al., 2008; Knaevelsrud and Simonsen, 2010; Komatsu and Ichimura, 2010; Rogov et al., 2014; Katsuragi et al., 2015; Valera et al., 2015; Marrero-Hernández et al., 2019).

Infection of macrophages with purified HIV-1 leads to a decrease in SQSTM1/p62, meaning that the autophagy flux is working during the first stages of the infection, and the lysosomal protease inhibitor pepstatin A reverts this situation (Campbell et al., 2015). This study was carried out in MDMs generated from blood of HIV seronegative donors. Another drug, LY 294002 that inhibits PIK3 class I as well as MTORC1, suppresses viral infection of macrophages in post-entry and post-reverse transcription steps prior to HIV-1 gene expression (François and Klotman, 2003). Pan-inhibitors of MTORC1 neutralize HIV-1 infection by interfering with viral entry in humanized mouse *in vivo* models, since these inhibitors decrease CCR5 levels at the cell-surface of target cells, and also act during viral transcription (Heredia et al., 2015; Akbay et al., 2020). Likewise, other research showed that HIV-1 gp41-induced apoptosis is mediated by caspase-3-dependent mitochondrial production of ROS (Garg and Blumenthal, 2006), which acts as a stimulus to sustain the autophagy process (Levonen et al., 2014; Filomeni et al., 2015). This phenomenon is inhibited by the HIV-1 protease inhibitor nelfinavir (Garg and Blumenthal, 2006).

Trehalose, a natural sugar that inhibits the cellular import of glucose and fructose, with concomitant stimulation of autophagy (Sarkar et al., 2007; Castillo et al., 2013; DeBosch et al., 2016; Mardones et al., 2016) has been found to be involved in controlling early HIV-1 infection (Rawat et al., 2020). Although its action on autophagy is not well elucidated (Yoon et al., 2017), it appears that knockdown of the autophagic *ATG5* gene reduces the anti-HIV-1 effect of trehalose in primary human macrophages (Rawat et al., 2020). Likewise, trehalose promotes viral material degradation by MTOR-independent autophagy soon after viral entry. It is noteworthy that trehalose also affects viral entry by down-regulating viral receptors CCR5 and CD4 in CD4^+^ T cells and macrophages (Rawat et al., 2020).

On the other hand, the ability of HIV-1 Env-early signal to promote autophagy has been confirmed by specific small interfering RNA (siRNA) silencing of *BECN1* and *ATG7* in CD4^+^ T lymphocytes, which induce a marked decrease in the Env-mediated cell death (Espert et al., 2006). Similar data have been reported in macrophages and microglia (Kyei et al., 2009; El-Hage et al., 2015). Of note, microglia are the only true CNS parenchymal macrophages and it is thought that they constitute 5-10% of total brain cells (Aguzzi et al., 2013; Li and Barres, 2018). Autophagy inhibition by *BECN1* silencing has also been proposed to mitigate neurodegenerative effects mediated by HIV-1 induced inflammation (El-Hage et al., 2015). In this line of action and by using large-scale RNAi screening, several autophagy- and lysosome-related genes have been found to be involved in HIV-1 replication, such as *ATG7*, *ATG12*, *ATG16L2 (autophagy related 16 like 2)*, *MAP1LC3B*, and *LAPTM5 (lysosomal protein transmembrane 5)* and *CLN3 (CLN3 lysosomal/endosomal transmembrane protein, battenin)*, respectively (Brass et al., 2008; Eekels et al., 2012), as well as *PIK3R4 (phosphoinositide-3-kinase regulatory subunit 4)*, *ATG4A (autophagy related 4A cysteine peptidase)*, *ATG5*, *ATG7* and *BECN1*, in cell lines or in cells isolated from blood samples (Kyei et al., 2009; Campbell and Spector, 2011, 2012a,b; Eekels et al., 2012). The importance of HIV-1 Env-mediated autophagy has also been identified in HIV-1 infection of CD4^+^ T cells as this process can selectively degrade the viral transactivator Tat, thus acting as an antiviral process. However, HIV-1 has evolved strategies to inhibit Env-induced autophagy in infected CD4^+^ T cells, thereby preventing Tat degradation and ensuring viral replication (Sagnier et al., 2015).

A recent study using cell lines suggests that ATG9 is required for HIV-1 infectivity in an Env-dependent way, as the knockout of *ATG9* gene does not affect Env incorporation into nascent virions, but it does have an effect on the infectivity, may be because ATG9 is thought to be a lipid transporter and perhaps promotes Env fusion with the host cell (Mailler et al., 2019). Similarly, HIV-1 Env induces a rapid shutdown of autophagy and immunoamphisomes in DCs, and is thus related to an increase in cell-associated HIV-1 and virus transfer to CD4^+^ T cells (Blanchet et al., 2010). This work reports that downregulation of autophagy by siRNA knock-down of *BECN1*, *ATG5*, and *MAP1LC3*, or by using 3-MA or chloroquine chemical inhibitors does not negatively affect the HIV-1 viral life cycle in cell lines. However, these treatments impede the innate and adaptive immune responses mediated by immunoamphisomes, in a similar way as HIV-1 does during HIV-1 infection in DCs isolated from donors or cell lines (Blanchet et al., 2010).

RNA silencing of the PIP_2_-membrane recycling driver, ARF6, or the use of mutant constructs of this protein inhibited HIV-1 Env-induced virus-cell membrane fusion, entry in and infection of CD4^+^ T cells, regardless of viral tropism, highlighting the importance of ARF6 in the regulation of HIV-1 infection (Barrero-Villar et al., 2008; Garcia-Exposito et al., 2011). Furthermore, inhibition of the actin-severing protein gelsolin results in HIV-1 gp120-mediated aberrant pseudopodia formation, a membrane dynamics process regulated by actin cytoskeleton reorganization, with aberrant viral receptors clustering at cell-surface that inhibit early HIV-1 infection (Garcia-Exposito et al., 2013). Other studies demonstrate that specific siRNA inhibition of *HDAC6* increases HIV-1 entry due to the increased amount of acetylated α-tubulin that favors virus-cell membrane fusion (i.e., pore fusion formation) and infection (Valenzuela-Fernandez et al., 2005; Valera et al., 2015).

CRISPR/Cas9, a promising technology in the development of strategies against different pathologies, has emerged as a potential anti-HIV-1 tool. One of the first factors targeted by this strategy is *TRIM5α*, which could be modified to restrict retroviral infection by promoting autophagy against HIV-1, soon after viral entry into the cell (Hatziioannou et al., 2004; Stremlau et al., 2004; Mandell et al., 2014a, b; Richardson et al., 2014; Ribeiro et al., 2016; Saha et al., 2020). Thus, it has been proposed that HSCs harvested from an HIV-positive patient could be transduced with an adeno-associated virus (AAV) vector bearing the Cas9 enzyme together with the single guide RNA (sgRNA) targeting *TRIM5α* and a repair template to introduce the mutations to target HIV-1 (Weatherley et al., 2017). These transgenic HSCs bearing the mutated *TRIM5α* would be implanted to achieve long-term cell repopulation in order to generate a durable subset of CD4^+^ T cells resistant to HIV-1 infection as reported with the *CCR5* gene (Xu et al., 2017). Editing of the *TRIM5α* gene was first reported in cell lines with suboptimal results (Dufour et al., 2018). However, this study suggested that the editing of the *TRIM5α* gene could be feasible in human cells. In this regard, biallelic CRISPR/Cas9-mediated editing of the *TRIM5α* gene has recently been associated with the protection of human T lymphocytic cells against infection by HIV-1 (Désaulniers et al., 2020). Furthermore, a link between TRIM5α and several autophagy factors, such as *BECN1*, *ATG7* and *ULK1*, has been observed by targeting these genes with the CRISPR/Cas9 editing machinery (Saha et al., 2020). This genetic depletion strategy inhibits the anti-HIV-1 proinflammatory function of TRIM5α impairing its capacity to activate adaptor related protein complex 1 (AP1) and NFKB factors that results in the inhibition of the production of the antiviral IFNB1 (named IFN-β) (Saha et al., 2020). All these data are indicative of the importance of the interplay between TRIM5α and autophagy during HIV-1 infection (Saha et al., 2020).

**FIGURE 2:**
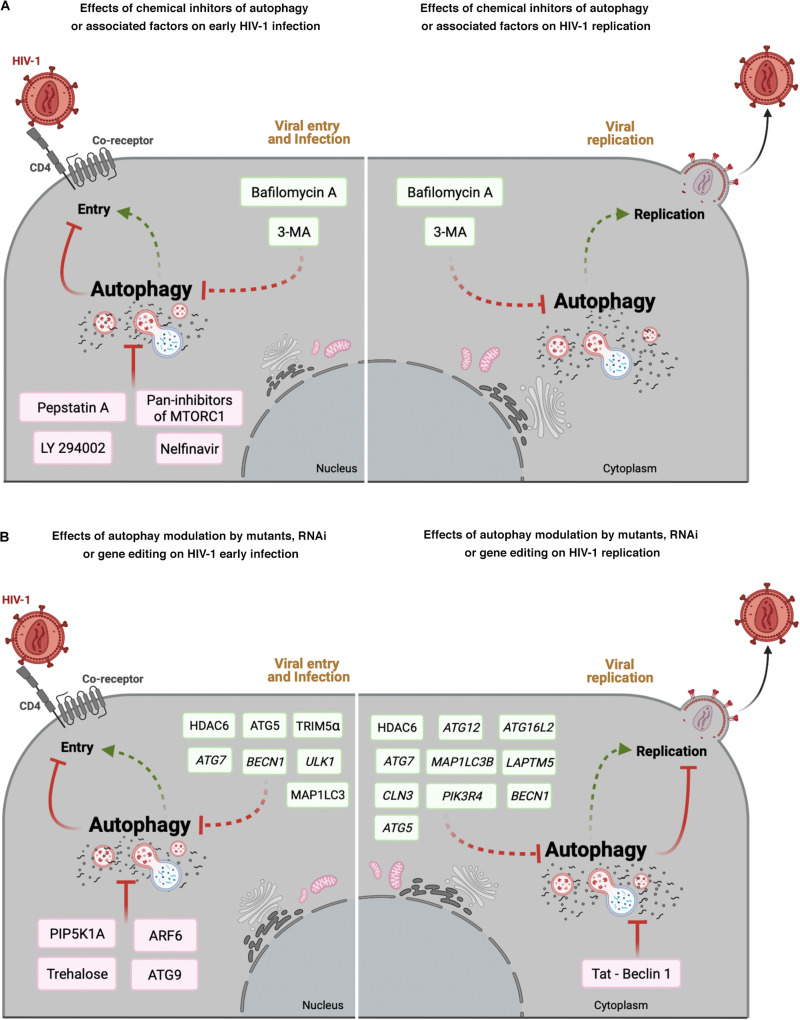
Effect of autophagy modulation on HIV-1 infection. Cell scheme summarizing the autophagy modulatory effect on HIV-1 viral life cycle (early steps: viral entry and infection; late steps: viral replication). **(A)** This panel shows that chemical inhibitors of autophagy and associated factors could activate (factors indicated in green boxes) or prevent (factors indicated in pink boxes) early HIV- 1 infection **(left)**, or promote viral replication **(right)**. **(B)** This panel shows the effect of mutants of autophagy associated factors, or specific RNAi or CRISPR/Cas editing acting on autophagy-associated genes in HIV-1 early infection or viral replication. Autophagic factors that activate HIV-1 infection or replication are indicated in green boxes, whereas factors inhibiting HIV-1 are indicated in pink boxes. Arrow colors represent the reported positive (green) and negative (red) effects on the different steps of the viral life cycle, with dotted arrows signaling the HIV-1 activation pathway. This figure supports the information summarized in Table 2. Cell schemes were created with BioRender.com.

Taken together, all the above studies (summarized in Table 2 and Figure 2), clearly show that autophagy is a potential therapeutic target to control HIV-1 infection and associated pathogenesis.

**TABLE 2:** Summary of the effect of autophagy modulation in the early steps of HIV-1 infection.

	Effect of autophagy modulation by chemical inhibitors* on HIV-1 viral cycle		Effect of autophagy-gene modification (mutants, RNAi or CRISPR/Cas) on HIV-1 viral cycle	
Effect on HIV-1	Viral entry and Infection	Viral replication	Viral entry and Infection	Viral replication
**+**	3-MA^B^, Bafilomycin A	3-MA, Bafilomycin A	*HDAC6*^2^, *ATG5*^2^, *ATG7*^A, 2, 3^, *BECN1*^A,2,3^, *MAP1LC3*, *TRIM5α*^3^, *ULK1*^3^	HDAC6^1^, *ATG7*^2^, *ATG12^2^, ATG16L2^2^, MAP1LC3B^2^, LAPTM5^2^, CLN3^2^, PIK3R4^2^, ATG4A^2^, ATG5^2^, ATG7^2^, BECN1^2^*
**-**	Pepstatin A^A^, LY 294002^A^, Pan-inhibitors of MTORC1, nelfinavir		PIP5K1A^1, 2^, ARF6^1,2^, Trehalose^A^, ATG9^B^	Tat-Beclin 1 construct^A^

The table summarizes the autophagy modulatory effect on HIV-1 viral cycle (viral entry and infection or viral replication) exerted either by chemical inhibitors acting on autophagy-associated factors, *which could activate or prevent autophagy, or by acting on autophagy-associated genes by using related mutants, specific RNA interference or CRISPR/Cas editing, as follows: ^1^ Dead mutant or modified protein; ^2^ RNAi; ^3^ CRISPR/Cas9. Left column groups the reported positive (**+)** and negative (**−**) effects on the viral cycle. In general, all these data refer to results obtained in CD4^+^ T lymphocytes, and when data have also been reported in other cell types and tissues, they are indicated as follows: ^A^Macrophages, ^B^DCs and ^C^studies in CNS (microglia). This information is illustrated in Figure 2.

## Concluding Remarks

One way to block HIV-1 transmission is to inhibit viral spreading into the immune host cells by acting on the viral machinery rather than targeting the intracellular enzymes. However, this is a complex goal, due to viral latency and because viral proteins are highly variable if one only considers the virus as a potential long-term drug-resistant quasi-species (Chun et al., 1998; Schuurman et al., 2002; Zhu et al., 2002; Korn et al., 2003; Belmonte et al., 2007; Buzon et al., 2014; Raymond et al., 2014; Ananworanich et al., 2016; Lorenzo-Redondo et al., 2016; Brenner et al., 2017; Malet et al., 2017; Bandera et al., 2019; Monaco et al., 2020). This is why many of the efforts in basic and clinical research have been aimed at developing strategies to block the virus entry into target cells. Most of the current approaches are based on pharmacological inhibition of the Env proteins, main receptor or co-receptors, as well as the membrane-hemifusion process before viral entry (reviewed in Mostashari Rad et al., 2018). The discovery of LTNP patients (Lambotte et al., 2005) may help to shed some light on identifying cellular mechanisms that are responsible for the control of the disease, not only because of their genetic background but also because of the characteristics of the virus.

Therefore, it is necessary to study the cellular events triggered by HIV-1 infection and to identify the factors implicated in the control of early HIV-1 infection in order to design valid therapies or vaccines. Hence, HIV-1 Env-mediated signals triggered in the cells susceptible to infection become a target of interest, since they induce cellular mechanisms involved in immune response (both innate and adaptive) (Blanco et al., 2003; Altfeld and Gale, 2015) among which is the autophagy process (Espert et al., 2006, 2007; Rotger et al., 2011; Nardacci et al., 2014, 2017). In addition, the recent discovery that functional Env from virus of VNP and RP individuals similarly triggers autophagy shows the importance of this mechanism in HIV-1 early infection and pathogenesis (Nardacci et al., 2014; Cabrera-Rodriguez et al., 2019), and this is supported by the fact that non-functional, signaling-defective primary HIV-1 Envs are associated with the LTNP-EC phenotype (Casado et al., 2018).

The duration of the HIV-1 Env-mediated signals on target cells differentially acts on autophagy functions, having distinct functional roles in HIV-1 infection and pathogenesis. Productive early HIV-1 infection requires the inhibition of autophagy and its related machinery, whereas non-productive signaling in bystander target cells promotes late autophagy and subsequent cell death (Blanco et al., 2003; Valenzuela-Fernandez et al., 2005; Espert et al., 2009; Casado et al., 2018; Cabrera-Rodriguez et al., 2019). In this respect, pro-autophagic and tubulin-deacetylase HDAC6 functions play a crucial role in limiting early HIV-1 entry and infection (Valenzuela-Fernandez et al., 2005, 2008; Valera et al., 2015), which is indicative of the relevance for HIV-1 of abrogating autophagy and factors related to allowing productive infection from the very beginning of HIV-1 fusion and entry. Other studies have analyzed the importance of autophagy after infection or in non-infected bystander cells in long contact with infected Env-expressing cells (Espert et al., 2006, 2007; Denizot et al., 2008; Shah et al., 2011), again confirming an interplay between autophagy and HIV-1 which is key for viral replication achievement and pathogenesis. It should be mentioned that some conclusions may depend on viral and cellular models used to look for this interplay that might bias the reported pro- or anti-autophagy mechanisms.

Moreover, viral factors that could impair or block the autophagy response to infection have also been looked for. In addition, it has been reported that Vif interacts with APOBEC3G promoting its degradation by the proteasome, thus eluding antiviral APOBEC3G activity (Valera et al., 2015). Vif exerts an anti-autophagy role, due to its interaction with MAP1LC3, and that the blockade of the degradative process by Vif is independent of its association with APOBEC3G (Borel et al., 2015). It has recently been demonstrated that Vpr is able to block HIV-1-mediated autophagy soon after its entry into CD4^+^ T cells (Alfaisal et al., 2019). Vpr down-regulates the expression of essential proteins, such as BECN1 or MAP1LC3, by inducing the proteasomal degradation of the transcription factor FOXO3 which control these autophagy genes. The power of Vpr in controlling autophagy is so strong that even in the presence of autophagy inducers, such as rapamycin or Torin 1, it is still able to block autophagy (Alfaisal et al., 2019).

All the aforementioned autophagy events in favor of and against early HIV-1 infection have led to a longstanding debate about the so-called “dual role” of autophagy in HIV infection. On the one hand, HIV-1 Env produces different signals that promote autophagy events which play a nullifying role in the virus life cycle but, on the other hand, other authors argue that the virus is able to activate or inactivate autophagy for its survival (reviewed in Espert et al., 2008; Killian, 2012; Leymarie et al., 2017). The results need to be carefully interpreted and different conclusions are reached because different strategies have been used, since the use of a construct of a protein, a virus or an Env expressed on a pseudovirus or at the cell-surface may not have the same effect on target cells, as the latter could also differ between independent studies. The case of what happens in the CNS of HIV-1 infected individuals is even more complicated as tissue can only be obtained post-mortem (Zhou et al., 2011; Dever et al., 2015) and the changes observed postmortem might not be present during life.

In summary, it is clear that autophagy has a highly important role in the early stages of HIV-1 infection, and deep characterization of its interplay with HIV-1 Env-triggered functions could play a major role in paving the way for HIV-1 eradication. Thus, more research is needed to address some important questions regarding the relevance of autophagy modulation (i.e., inhibition or activation) by the HIV-1 Env-mediated signaling during the early steps of the viral productive infection or in a context of long virus-target cell contact without productive infection but resulting in bystander cell death (i.e., a critical process that could favor immunodeficiency) (Espert et al., 2006, 2007, 2008, 2009). Elucidating the relationship between HIV-1 Env-mediated signals and autophagy is a priority, after observing that LTNP-EC phenotype is associated with non-functional Env unable to overcome the barrier of autophagy associated factors, such as HDAC6 restriction factor (Valenzuela-Fernandez et al., 2005; Casado et al., 2018; Cabrera-Rodriguez et al., 2019).

Similarly, controlled clinical trials are needed to determine whether addition of autophagy inducers together with ART in HIV-infected individuals may impact HIV-1 reservoirs *in vivo*. Hence, it is important to analyze the potential universal therapeutic modulation of autophagy in different HIV^+^ patients, considering factors such as age, coinfections and pharmacological interaction of autophagy drugs with ART. Likewise, there is a growing interest in understanding the complex relationship existing between autophagy functions and HIV-1 pathogenesis in treated patients co-exposed to illicit drugs (Cao et al., 2017). It seems that autophagy is targeted by the use of drugs in HIV^+^ patients. Thus, the abuse of drugs could favor viral replication not only by increasing inflammation, oxidative and ER stress, but also acting on autophagy (Qi et al., 2011; Dever et al., 2015; El-Hage et al., 2015), thereby affecting the efficiency of ART. On the other hand, autophagy inhibitors could also favor HIV-1 pathogenesis in this scenario, since it has been reported that autophagy inhibitors exacerbated HIV-1 Env/methamphetamine-mediated cell death (Cao et al., 2016). Hence, these data suggest a protective role of autophagy in astrocyte death. Moreover, the use of the autophagy process as a target for HIV-1 therapy should be carefully and deeply studied as this cellular mechanism is implicated in the development of pathogenic reactions, such as cancer or CNS disorders (Aita et al., 1999; Qu et al., 2003; Yue et al., 2003; Ravikumar et al., 2004; Iwata et al., 2005; Hara et al., 2006; Komatsu et al., 2006, 2010; Sarkar et al., 2007; Cherra and Chu, 2008; Koike et al., 2008; Pickford et al., 2008; Martinet et al., 2009; Guo et al., 2011, 2013; Yang et al., 2011, 2014; White, 2012; Levy et al., 2014; Vogl et al., 2014; Wei et al., 2014; Perera et al., 2015; Rohatgi et al., 2015; Tang et al., 2015; Bar-Yosef et al., 2019).

Therefore, it is important to understand how the interplay between autophagy and HIV-1 Env-mediated signal and infection could determine the clinical outcome of HIV-1 patients (i.e., comparing long-term non-progressors that control viremia with progressors) (Espert et al., 2006, 2007, 2008, 2009; Valenzuela-Fernandez et al., 2008; van Grol et al., 2010; Valera et al., 2015; Casado et al., 2018, 2020; Cabrera-Rodriguez et al., 2019) and pathogenesis. In sum, determining the mechanisms underlying HIV/autophagy interplay is probably key to developing novel drug candidates in the challenge of preventing HIV infection and related pathology, thereby offering new insights into HIV-1 functional cure and/or eradication.

## Author Contributions

A-VF and R-CR drafted and wrote the manuscript. All authors read, edited, supervised the scientific content and approved the final manuscript.

## Conflict of Interest

JB is founder and shareholder of AlbaJuna Therapeutics, S.L. The remaining authors declare that the research was conducted in the absence of any commercial or financial relationships that could be construed as a potential conflict of interest.
